# GlnR-Mediated Regulation of Short-Chain Fatty Acid Assimilation in *Mycobacterium smegmatis*

**DOI:** 10.3389/fmicb.2018.01311

**Published:** 2018-06-22

**Authors:** Xin-Xin Liu, Meng-Jia Shen, Wei-Bing Liu, Bang-Ce Ye

**Affiliations:** ^1^Laboratory of Biosystems and Microanalysis, State Key Laboratory of Bioreactor Engineering, East China University of Science and Technology, Shanghai, China; ^2^Collaborative Innovation Center of Yangtze River Delta Region Green Pharmaceuticals, College of Pharmaceutical Sciences, Zhejiang University of Technology, Hangzhou, China

**Keywords:** propionyl-CoA/acetyl-CoA synthetase, GlnR, short-chain fatty acid, nitrogen metabolism, post-translational modification, acylation

## Abstract

Assimilation of short-chain fatty acids (SCFAs) plays an important role in the survival and lipid biosynthesis of *Mycobacteria*. However, regulation of this process has not been thoroughly described. In the present work, we demonstrate that GlnR as a well-known nitrogen-sensing regulator transcriptionally modulates the AMP-forming propionyl-CoA synthetase (MsPrpE), and acetyl-CoA synthetases (MsAcs) is associated with SCFAs assimilation in *Mycobacterium smegmatis*, a model *Mycobacterium*. GlnR can directly activate the expression of *MsprpE* and *Msacs* by binding to their promoter regions based upon sensed nitrogen starvation in the host. Moreover, GlnR can activate the expression of lysine acetyltransferase encoding *Mspat*, which significantly decreases the activity of MsPrpE and MsAcs through increased acylation. Next, growth curves and resazurin assay show that GlnR can further regulate the growth of *M. smegmatis* on different SCFAs to control the viability. These results demonstrate that GlnR-mediated regulation of SCFA assimilation in response to the change of nitrogen signal serves to control the survival of *M. smegmatis*. These findings provide insights into the survival and nutrient utilization mechanisms of *Mycobacteria* in their host, which may enable new strategies in drug discovery for the control of tuberculosis.

## Introduction

Intracellular nutrient utilization is a very important survival mechanism for pathogenic *Mycobacteria* such as *Mycobacterium tuberculosis* (*Mtb*) within the human host. In the host of macrophages, *Mtb* grows preferentially in phagosomes, a known nutritionally-constrained environment, where *Mtb* can exploit fatty acids and cholesterol as carbon and energy sources to survive (Pandey and Sassetti, 2008). In previous works, multiple lines of evidence support the idea that host fatty acids and cholesterol rather than carbohydrates serve as vital carbon sources for *Mtb* during infection and latency. Since the 1950s, *Mtb* was found to propagate in mammalian tissues preferentially by assimilating fatty acids as nutrients (Bloch and Segal, 1956). Thereafter, plenty of researches have revealed that the metabolism of fatty acid facilitate *Mtb* survival during infection (McKinney et al., 2000; Muñoz-Elías et al., 2006; Marrero et al., 2010). In addition, *Mtb* can also uptake cholesterol which is a crucial nutrient for *Mtb* survival within macrophages (Van der Geize et al., 2007; VanderVen et al., 2015) as well as various *in vivo* infection models (Chang et al., 2007; Pandey and Sassetti, 2008; Yam et al., 2009; Hu et al., 2010; Nesbitt et al., 2010).

In *Mtb*, fatty acids and cholesterol are degraded to short chain fatty acids (SCFAs, such as acetate and propionate) to be assimilated to generate energy or synthesize lipid (Yang et al., 2009; Wipperman et al., 2014). So far, assimilation of SCFAs and cholesterol is considered as a characteristic of *Mtb* (Cole et al., 1998). In brief, the majority of acetyl-CoA enters the TCA cycle to provide energy, and the remainder is used to synthesize mycolic acid through malonyl-CoA. Propionyl-CoA is assimilated into central carbon metabolism via the methylcitrate cycle and the methylmalonyl pathway (Savvi et al., 2008; Griffin et al., 2012). However, accumulation of propionyl-CoA is toxic and inhibitory to a few pivotal metabolic enzymes. Therefore, the metabolism of propionyl-CoA need to be tightly controlled (Carter and Alber, 2015). One known pathway for propionyl-CoA reduction is the synthesis of lipids such as phthiocerol dimycoserate (PDIM) (Wipperman et al., 2014), which contributes to sustaining infection and virulence. Consequently, the assimilation of SCFAs play a crucial role for the survival and virulence of *Mtb*.

In bacteria, SCFAs are assimilated via acyl-CoA intermediates which are generated by acyl-CoA synthetases catalysis, such as acetyl-CoA synthetase (Acs) and propionyl-CoA synthetase (PrpE). These enzymes were first characterized in *Salmonella* Typhimurium (Horswill and Escalante-Semerena, 1999). As a common route for propionyl-CoA synthesis in microorganisms, PrpE has also been investigated in other bacterial species (Rajashekhara and Watanabe, 2004; Kang et al., 2012; Yang et al., 2014). In addition to PrpE, Acs could also act as a secondary route for propionyl-CoA synthesis (Liu et al., 2014). Previous work has shown that the *prpE* deletion mutant could still use propionate as carbon and energy sources, since Acs could compensate for the lack of PrpE (Horswill and Escalante-Semerena, 1999).

During the assimilation of SCFAs, PrpE, or Acs are subject to regulation at multiple levels. On the transcriptional level, the regulatory mechanism of *prpE* is well understood in Gram-negative bacterium *Escherichia coli*, in which *prpE* is regulated by PrpR, a transcriptional activator for *prpBCDE* operon. Furthermore, similar phenomena were also found in *Salmonella* Typhimurium and *Mtb* (Palacios and Escalante-Semerena, 2000; Liu et al., 2014; Masiewicz et al., 2014). For *acs*, many direct and indirect regulators have been identified and studied in *E. coli*, these regulators can regulate the transcription of *acs*. For example, catabolite repression and the nucleoid proteins Fis and IHF act as direct factors, while several other indirect transcription factors were also identified (Kumari et al., 2000; Wolfe, 2005; Bernal et al., 2016). Meanwhile, in Gram-positive bacterium *Bacillus subtilis*, the *acs* gene is controlled by GTP-sensing transcription factor CodY and carbon flux regulator CcpA (Warner and Lolkema, 2003; Starai and Escalante-Semerena, 2004; Wolfe, 2005). For *Mtb*, LucA was identified recently as a lipid uptake coordinator to regulated the assimilation of both fatty acids and cholesterol when *Mtb* grow in macrophages (Nazarova et al., 2017).

Except for transcriptional modulation, post-translational modification (PTM) is an efficient mechanism for regulating gene expression and enzymatic activity in response to changing physiological conditions (Gardner et al., 2006). PrpE was found to undergo propionylation *in vivo* which inactivates its activity, while Acs is the first and best-known enzyme modulated by reversible lysine acetylation (RLA) in bacteria (Hayden et al., 2013). Recently, many scientists studied the mechanism of RLA regulating the activity of Acs in various bacteria, such as *E. coli, Salmonella enterica*, and *Mtb* (Starai et al., 2002; Castaño-Cerezo et al., 2011; Noy et al., 2014). Similar to Acs, PrpE of *S. enterica* is also regulated by reverse propionylation (Garrity et al., 2007). In *Mtb*, metabolism of fatty acid was found to be mediated by lysine acylation (Nambi et al., 2013). So far, much research progress has been made on the regulation of SCFAs assimilation, such as bacterial propionyl-CoA synthesis pathways. However, most of them concentrate on *E. coli* and *Salmonella*, whereas little is known in mycobacteria.

In this study, we scrutinized the regulatory mechanism of SCFAs assimilation in *M. smegmatis* at the transcriptional and post-translational levels. We found that important SCFA metabolic enzymes, i.e., propionyl-CoA synthetase (MsPrpE) and four AMP-forming Acs, are all activated at the transcriptional level by nitrogen-sensing regulator GlnR under nitrogen limited conditions. On the contrary, with nitrogen starvation, GlnR activates the expression of acetyltransferase coding gene (*Mspat*). Next, the highly expressed MsPat increase the acylation level of MsPrpE and MsAcs, resulting in significant decrease in enzymatic activity. Consequently, the results reveal that GlnR senses the change in environmental nitrogen signal to regulate the assimilation of SCFAs at the transcriptional and post-translational levels. These regulations directly impact the growth of *M. smegmatis* which utilizes different SCFAs and cholesterol as carbon sources. These findings provide insights into the mechanisms of SCFA assimilation, which affects the survival, detoxification, and infection of *Mycobacteria*.

## Materials and Methods

### Bacterial Strains and Growth Conditions

The bacterial strains and plasmids used in this work are listed in **Table 1**. *Mycobacterium smegmatis* mc^2^ 155 wild type and its *glnR* deleted mutant (*ΔglnR*) were grown in LB broth (supplemented with 0.05% Tween 80) at 37°C, 200 rpm or on LB agar plates at 37°C. All *E. coli* strains were cultured in LB broth or on LB agar. When needed, *M. smegmatis* was cultured in Sauton’s medium [3.6 mM KH_2_PO_4_, 2 mM MgSO_4_, 10 mM citric acid, 0.1 mM ferric citrate, 6 μM ZnSO4, 0.2% glycerol, 0.015% Tyloxapol (pH7.0)] (Jenkins et al., 2013), supplemented with (NH_4_)_2_SO_4_ at 1 mM (nitrogen limiting) or 30 mM (nitrogen excess) after being washed twice in nitrogen-free medium. For growth curves, strains were grown in M9 media containing 12.6 mM Na_2_HPO_4_, 22 mM KH_2_PO_4_, 8 mM NaCl, 19 mM NH_4_Cl, 2 mM MgSO_4_, 0.1 mM CaCl_2_, and 0.1% tyloxapol (Hayden et al., 2013), and the starting OD_600_ was 0.06. Provided carbon sources included 10 mM glucose or 10 mM sodium acetate, or 10 mM sodium propionate.

**Table 1:** Strains and plasmid used in this study.

Strains and plasmid	Source or reference
*Mycobacterium smegmatis mc^2^ 155*	Jenkins et al., 2012
*Mycobacterium smegmatis mc^2^ 155 ΔglnR*	Jenkins et al., 2012
*Mycobacterium smegmatis mc^2^ 155 ΔglnR::glnR*	In this study
*Escherichia coli DH5α*	Novagen
*Escherichia coli BL21(DE3)*	Novagen
pET-28a	Thermo Scientific
pMV261	Stover et al., 1991
pMV361	Stover et al., 1991

The *glnR* complemented strain was constructed referring to a previous work (Stover et al., 1991). In brief, *glnR* gene was amplified by PCR. The amplicon was inserted into shuttle vector pMV361, which was transformed into *E. coli DH5α* for recombinant vector screening and confirmation by PCR and sequencing. The verified plasmids were introduced into *ΔglnR* competent cell by electric-mediated protoplast transformation. Transformants were selected by its resistance to kanamycin and subsequently analyzed by qRT-PCR to verify integration of *glnR* gene into the *M. smegmatis* genome.

### Cloning, Over-Expression, and Purification of Proteins

*Msmeg_5784 (glnR), Msmeg_5458, Msmeg_6179 (MsacsA1), and Msmeg_5404 (MsprpE)* genes were amplified from the genomic DNA of *M. smegmatis*, and *Rv0818* was amplified from the genomic DNA of *Mtb* using a seamless cloning and assembly kit. The PCR products were homologous recombinated with pET-28a which has been digested with EcoRI and HindIII to generate a recombinant vector. The clone was confirmed by PCR and sequencing. The protein was expressed in *E. coli BL21 (DE3)* strain. A single colony was inoculated into 5 mL of LB medium containing 1% kanamycin and cultured at 37°C and 200 rpm overnight. The activated bacteria were then transferred to 50 mL of LB medium and induced with IPTG (0.7 mM) overnight until the OD_600_ reached 0.6.

For protein expression, cells were harvested by centrifugation (8000 rpm, 10 min) and washed twice with PBS (pH8.0). The cells were suspended by 25 mL of PBS and sonicated. The debris was separated from the soluble fraction by centrifugation at 8,000 rpm and 4°C for 30 min. Supernatant was purified using Ni-NTA agarose column (Merck, Darmstadt, Germany). The column was washed with 10 mL of 10 mM imidazole in 50 mM NaH_2_PO_4_ and 300 mM NaCl, pH 8.0. The desired protein was eluted with 20–250 mM imidazole in 50 mM NaH_2_PO_4_ and 300 mM NaCl (pH 8.0). We analyzed the fractions by SDS-PAGE and polled the ones that contained the desired protein. The polled fractions were dialyzed against PBS containing 10% glycerol. The *his* tag of MsAcs and MsPrpE was removed by thrombin treatment at 4°C overnight. The protein was concentrated using centrifugation with a 30 K cutoff (Millipore, Billerica, MA, United States). All protein concentrations were measured using the BCA quantitative kit.

### Over-Expression of His-Acs, His-GlnR, and His-PrpE From *M. smegmatis*

*Msmeg_5784 (glnR), Msmeg_6179 (MsacsA1)*, and *Msmeg_5404 (MsprpE)* genes were amplified from the genomic DNA of *M. smegmatis* by PCR and then cloned into the shuttle vector pMV-261 which has the resistance of kanamycin yielding -5784, pMV-261-6179 and pmv-261-5404 (Stover et al., 1991). The overexpression plasmids of pMV-261-5784, pMV-261-6179, and pMV-261-5404 were confirmed by PCR and sequencing were introduced into *M. smegmatis* by electric-mediated protoplast transformation. The complementary strain of His-GlnR was obtained by introducing pMV-361-5784 recombined vector to *glnR* knockout (*ΔglnR*) strain. The overexpression *M. smegmatis* strains were obtained by kanamycin resistance screening and confirmed by protein SDS-PAGE.

The overexpression strains were harvested by centrifugation (8000 rpm, 10 min) and washed twice with PBS (pH8.0). The cells were resuspended in PBS and sonicated, and the debris were removed by centrifugation at 8,000 rpm 4°C for 30 min. The desired protein was purified using Ni-NTA agarose column as mentioned above. Samples were analyzed using SDS-PAGE and then subjected to Western blot analysis. We used two antibodies: HRP conjugated acetyl-lysine antibody (Cat# ICP0380) (Immunechem Pharmaceuticals, Burnaby, BC, Canada), and HRP conjugated His tag antibody (Abmart). The bound antibody was visualized using enhanced chemiluminescence method.

### Electrophoretic Mobility Shift Assay (EMSA)

The putative promoter regions (upstream region about -300–60) containing GlnR-binding sites were amplified by PCR using the primers listed in **Table 2**, then biotin-labeled by PCR with biotin-modified universal primer (5′ biotin-AGCCAGTGGCGATAAG-3′) (Liao et al., 2014). The PCR products were identified by agarose gel electrophoresis and purified using a PCR purification kit (Shanghai Generay Biotech), and were then used as EMSA probes. The concentrations of probes were quantified with a microplate reader (Biotek, United States) and diluted to 5 ng/μL. EMSAs assay was carried out using the Chemiluminescent EMSA Kit (Beyotime Biotechnology, China). The binding solution (total volume was 10 μL) containing 1 μL probes, varying amounts of purified GlnR and 1 μL Gel-Shift binding buffer (50 μg/mL poly (dI-dC), 1 mM DTT, 10% glycerol, 1 mM EDTA, 50 mM NaCl, 25 mM MgCl_2_, 10 mM Tris–HCl, pH 8.0, 0.01% Nonidet P40) was incubated at room temperature for 20 min. After binding, the samples were separated on a non-denaturating PAGE gel in ice-bathed 0.5 × Tris-borate-EDTA (Shanghai Bioscience) at 170 V. Then the probe, protein and their compounds were transferred to a nylon membrane at 160 V for 30 min. Finally, the bands were detected by BeyoECL Plus (You et al., 2017).

**Table 2:** Probes used in the Electrophoretic mobility shift assay (EMSA) assay.

Name	Sequence (5′–3′)
MSM5650P-F	AGCCAGTGGCGATAACTCGTCC CGAAGAACTCCGT
MSM5650P-R	AGCCAGTGGCGATAAGGCAGCTAT GCTCTACGTCACAA
MSM5404P-F	AGCCAGTGGCGATAACCACGATCA GGTCGTATCCG
MSM5404P-R	AGCCAGTGGCGATAAGACTCGAAC AAGGCACGGTAA
MSM6179P-F	AGCCAGTGGCGATAAGCGACGAGGC CACGCAGATC
MSM6179P-R	AGCCAGTGGCGATAAGCCCAGTCCC AAAATTTCCAACA
MSM3986P-F	AGCCAGTGGCGATAAGCCACCGGAC CCGCTACGA
MSM3986P-R	AGCCAGTGGCGATAAGCCTGCATCA GCCCTGCCATAT
MSM0718P-F	AGCCAGTGGCGATAAGGCCCGATAC CAGCACCACG
MSM0718P-R	AGCCAGTGGCGATAAGGTCACAGTC CTAACACACCAGCC
MSM5458P-F	AGCCAGTGGCGATAAGGCGCTCAAA AGCCGCCTC
MSM5458P-R	AGCCAGTGGCGATAAGGCGCTCAA AAGCCGCCTC
RV3667P-F	AGCCAGTGGCGATAAGGCCAACGAA CGACGCCACA
RV3667P-R	AGCCAGTGGCGATAAGCAGGATGCA GTCATAGCCAAGAAA
RV0998P-F	AGCCAGTGGCGATAAGCAGCCGG TGATGATCAGACTCATG
RV0998P-R	AGCCAGTGGCGATAAGTGCCCG CCGATGCGCTAC

### Quantitative Real-Time PCR

Cells grown to exponential stage in nitrogen-limited medium were harvested by centrifugation at 4°C. Total RNA was prepared using RNeasy Mini Kit (Qiagen, Valencia, CA, United States). The RNA quality was analyzed by 1% agarose gel electrophoresis, then the RNA was quantified by microplate reader (BioTek, United States). The residual DNA was digested and RNA was reverse transcribed using a PrimeScript^TM^ RT reagent Kit with gDNA Eraser (Takara, Shiga, Japan) for qRT-PCR. PCR reactions were performed using 2 × RealStar Green Fast Mixture (GenStar) with primers listed in **Table 3**. Each PCR reaction with 20 μL final volume contained 50 ng cDNA, and was performed on a CFX96 Real-Time System (Bio-Rad). The qPCR conditions were: 95°C for 5 min followed by 40 cycles of 95°C for 10 s, 60°C for 15 s, and 72°C for 30 s, and final extension at 72°C for 10 min (You et al., 2016).

**Table 3:** Primes used for qPCR.

Name	Sequence (5′–3′)
MSM3757RT-F	TGGTGTAGCGGTGGAATG
MSM3757RT-R	CGTTTACGGCGTGGACTA
MSM6179RT-F	TGGCGTTCTGGGAGAAGCA
MSM6179RT-R	CCTTGAGTTCGGCGTAGGTGAT
MSM5650RT-F	ACCACCAGCAAGCCCAAGC
MSM5650RT-R	CGGCGGTAGTTGTAGACGAAGAT
MSM0718RT-F	TGGACCGAACCGCTGACACAT
MSM0718RT-R	GCCGACCTCTGACAAGCCGTAA
MSM3986RT-F	TATGGCGGGTGATGGAA
MSM3986RT-R	CGTAGTGATCTCGGACAAGGA
MSM5404RT-F	ACTGGTGGCAGACCGAGACA
MSM5404RT-R	AACGCCGACAGGTACGAGGA
MSM5458RT-F	AGCAGCGTCAGCATCTCCGA
MSM5458RT-R	AGACGATCAGAGCACCCATCAAGA
MSM5784RT-F	TTCCCTGTTGGAGACGGGTAGTG
MSM5784RT-R	CACCGAGTGTGATCTTGCCTACATT

### *In Vitro* Protein Propionylation and Acetylation Assays

Propionylation assays were carried out in a 100 μL mixture that included buffer B (50 mM HEPES, pH 7.5), 2 μM MsAcs or MsPrpE, 20 μM Propionyl-CoA, and 2.7 μM MsPat with or without 1 mM cAMP. Acetylation assays were carried out in a 100 μL reaction mixture that contained buffer A (50 mM HEPES and 150 mM NaCl, pH 7.5), 2 μM MsAcs or MsPrpE, 100 μM Acetyl-CoA, and 2.7 μM MsPat with or without 1 mM cAMP. Reactions were incubated at 37°C for 2 h (Xu et al., 2011; Liu et al., 2016). After propionylation or acetylation, the MsPrpE or MsAcs was extracted from the reaction products using gel filtration and ultrafiltration (Xu et al., 2014). The propionylated or acetylated proteins were divided into two portions: one was used in SDS-PAGE Western blot to monitor the propionylation or acetylation, and the other was used to measure the change of enzymatic activity of MsPrpE or MsAcs (You et al., 2017).

### Western Blot Assays

The concentration of the protein was measured using BCA Protein Assay Kit. Samples were isolated by SDS-PAGE (10% acrylamide) and then transferred to a PVDF membrane at 380 mA for 1 h. The PVDF membrane was blocked in BSA blocking buffer for 2 h and then incubated with acetyl-lysine antibody (dilution of 1:15,000) at 4°C overnight. The blot was washed three times (once 10 min) with TBST buffer (20 mM Tris–HCl, pH 7.5, 150 mM NaCl, and 0.1% Tween 20), and the bound antibody was detected by ECL system (CTB, United States).

### *In Vitro* MsPrpE and MsAcs Assays

MsPrpE catalyzes the first step of propionate metabolism by converting propionate to propionyl-CoA. The formated propionyl-CoA was measured using a biosynthetic assay (Liu et al., 2014). The reaction mixtures contain 50 mM Tris–HCl (pH 8.0), 100 mM sodium propionate (sodium acetate), 5 mM ATP, 3 mM MgCl_2_, 0.5 mM DTT, 300 mM hydroxylamine, and 2.5 mM CoA in a total volume of 200 μL. MsPrpE or MsAcs was added into the mixture and incubated at 37°C. The color agent containing 0.2 M TCA, 0.4 M FeCl_3_6H_2_O, 5% (v/v) concentrated hydrochloric acid was added into the mixture, then incubating for 30 min to stop the reaction. The color generated was measured at 540 nm by microplate reader. The activity of MsPrpE was represented by the formation of propionylhydroxamate per protein amount and time (You et al., 2016).

### Resazurin Assays

Resazurin was used to monitor the growth and viability of *M. smegmatis* in different carbon resources. Strains were washed twice in media without carbon, and then growing in M9 media with 10 mM glucose, 10 mM acetate, 10 mM propionate, 10 mM cholesterol, or no carbon. The initial OD of strains is 0.05, after cultured at 37°C and 100 rpm for 72 h. The culture was transferred into a 96-well plate. Resazurin solution (12.5 mg/ml final concentration; Sigma) was then added, and incubated for 10 h. Emitted fluorescence signal at 590 nm was recorded. After 10 h of resazurin addition, the values were normalized to the wild-type strain under the same growth condition. Meanwhile, samples without cells and with cells but without carbon source were used as negative controls (Hayden et al., 2013).

### Mass Spectrometry Analysis of the Acylation Site

The acetylated and propionylated sites of MsAcsA1 and MsPrpE were analyzed by mass spectrometry (MS) according to our previous work (Liu et al., 2016). In brief, acetylated or propionylated MsAcsA1 or MsPrpE was analyzed by SDS-PAGE. The single protein band corresponding to MsAcsA1 or MsPrpE was cut from the gel and washed twice with 50% ethanol to remove the stain. Gel pieces were rehydrated using 10 ng/μL trypsin in 50 mM ammonium bicarbonate (Promega, Madison, WI, United States), and digestion was continued at 37°C for 12 h. Then, the digests were analyzed by LC-MS/MS. Full MS spectra (m/z range: 350–1700) were obtained in the resolution of 240,000. The five most intense ions were extracted in turn from each full MS spectrum for MS/MS fragmentation. Automatic gain control (AGC) setting for ion trap and orbitrap was 3E4 and 1E6. A Mascot search engine (v2.3.01, Matrix Science, United Kingdom) was used to check against the MsAcsA1 or MsPrpE sequence from “.mgf” files generated by Thermo Proteome Discoverer (Thermo Fisher Scientific). Within the searching process, these parameters were used with 10 ppm peptide mass tolerance, 0.5 Da fragment mass tolerance, and selected charge states (+2, +3, and +4). The program-identified peptides were verified manually.

## Results

### GlnR Regulates Gene Expression in SCFA Assimilation in Response to Nitrogen Condition

**FIGURE 1:**
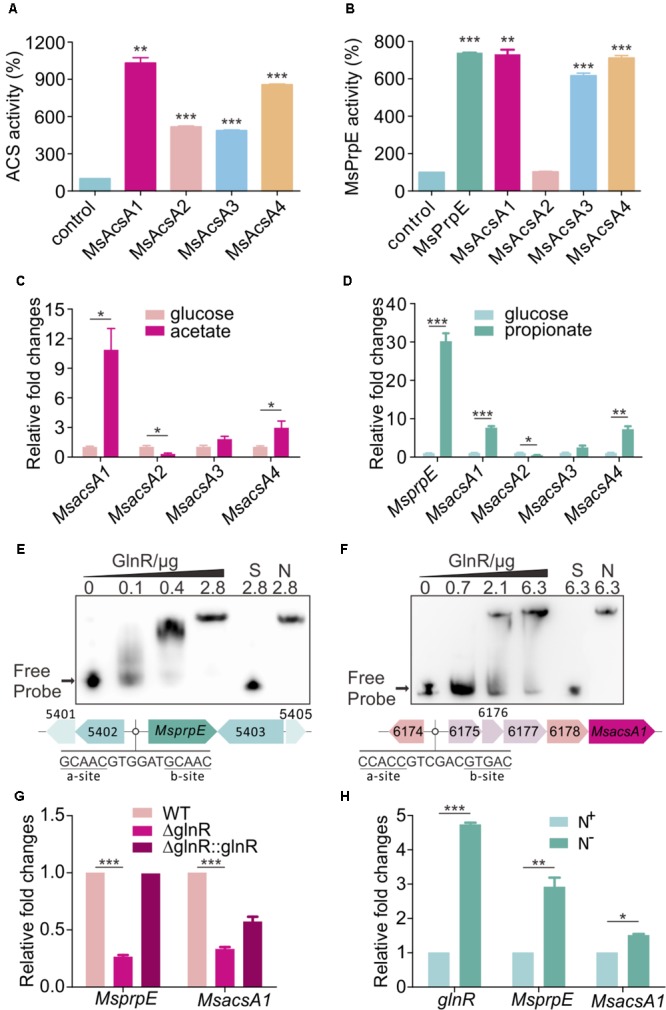
GlnR regulates the expression of *Msacs* and *MsprpE* in *Mycobacterium smegmatis*. **(A)** Acetyl-CoA synthetase activity of MsAcs. **(B)** Propionyl-CoA synthetase activity of MsPrpE and MsAcs. **(C)** Transcriptional levels of the *Msacs* genes in wild type (WT) strain grown in glucose or acetate as carbon sources. **(D)** Transcription levels of *MsprpE* and *Msacs* in WT grown in glucose or propionate as carbon sources. **(E,F)** Electrophoretic mobility shift assay (EMSA) identification of GlnR binding with the promoter region of *MsprpE* and *MsacsA1*. GlnR protein labeled with His tag incubate with probe (5 ng, MsprpE or MsacsA1). Unlabeled specific probe (S) (200-fold excess) or non-specific competitor DNA (sperm DNA) (N) were used as controls. **(G)** Differential expression analysis of *glnR* regulating *MsprpE* and *MsacsA1*. WT, Δ*glnR* (*glnR* knocked out mutant) and Δ*glnR*::*glnR* (*glnR* complementary) were cultured at 30°C to the middle exponential phase. **(H)** WT was cultured in Sauton medium supplemented with N^+^ and N^-^, following comparative analysis of *glnR, MsprpE*, or *MsacsA1* gene in the excess (N^+^) or limited (N^-^) nitrogen condition. Error bars represent the standard error, *n* = 3. *P*-values were determined by two-tailed unpaired *t*-test. ^∗^*P* < 0.05; ^∗∗^*P* < 0.01; ^∗∗∗^*P* < 0.001.

Through *in silico* analysis of the SCFAs metabolic pathway in *M. smegmatis*, we found one AMP-forming propionyl-CoA synthetase (MsPrpE, MSMEG_5404, EC:6.2.1.17) and four AMP-forming Acs (EC:6.2.1.1, hereinafter referred to MsAcsA1, MSMEG_6179; MsAcsA2, MSMEG_0718; MsAcsA3, MSMEG_3986; MsAcsA4, MSMEG_5650). For *in vitro* MsAcs assays, we found that all MsAcs had Acs activity (**Figure 1A**), and the activity of MsAcsA1 was highest. Meanwhile, MsPrpE, MsAcsA1, MsAcsA3, and MsAcsA4 also showed high propionyl-CoA synthetase activity (**Figure 1B**). In order to analyze the expression of *Msacs* and *M*s*prpE* genes in response to different fatty acids in *M. smegmatis*, we investigated the transcription activity of four *Msacs* and one *MsprpE* genes in cells grown in acetate and propionate using glucose as control. As seen in **Figure 1C**, the expression of *MsacsA1* was the highest when acetate was the sole carbon source, which was consistent with the ACS activity in **Figure 1A**. Therefore, this result indicates that *MsacsA1* could play a major role in acetate assimilation. In the uptake of propionate (**Figure 1D**), the *MsprpE* gene was highly expressed. Combining the enzymatic activity data in **Figure 1B**, it is suggested that *MsprpE* was responsible for propionate uptake. At the same time, *MsacsA1* and *MsacsA4* also played an important role in propionate metabolism. As AMP-forming Acs, MsAcsA1 is essential for *M. smegmatis* (Xu et al., 2011; Hayden et al., 2013; Chopra et al., 2014). Based upon existing data on acetate metabolism, we selected *MsAcsA1* as a representative *Msacs* gene combining *MsprpE* to explore the mechanism of SCFA assimilation.

Using MEME/MAST and PREDetector to analyze the motif of *MsprpE* and *Msacs* genes (Liao et al., 2014), we found a classic GlnR binding box in the promoter region of these genes which consists of one a site and one b site separated by a six-nucleotide motif (a-n6-b) (Amon et al., 2009; Liao et al., 2014). In order to validate the binding of GlnR to this box, purified GlnR protein was diluted to different concentrations for the EMSA assay using a DNA probe containing those sites. The unlabeled specific probe (S) (200-fold excess) or non-specific competitor DNA (sperm DNA) (N) were used as controls. Results indicated that GlnR could bind with the promotor region of the *MsprpE* and four *Msacs* genes in *M. smegmatis* (**Figures 1E,F** and **Supplementary Figure S1A**). To investigate the regulation of GlnR to *MsprpE* and *Msacs* genes, we constructed a *glnR* deletion strain of *M. smegmatis* (*ΔglnR*) and *glnR* complementary strain (*ΔglnR::glnR*) for qPCR assay and analyzed the differential expression of those genes in the mutant strains. As shown in **Figure 1G** and **Supplementary Figure S1B**, the transcriptional level of *prpE* and all four *Msacs* genes sharply decreased compared to those in the wild type (WT). Meanwhile, the expressions of these genes were recovered in *ΔglnR::glnR*. The qPCR and EMSA results indicated that the *MsprpE* gene and the four *Msacs* genes were all directly subject to the regulation of GlnR in *M. smegmatis*. GlnR is known as a globe nitrogen regulator, whose expression is activated in nitrogen limited environments (Amon et al., 2008). To test whether nitrogen availability interferes with the regulation of GlnR on *MsprpE* and *Msacs*, we established a comparative model with Sauton medium supplementing excess (N^+^) or limited (N^-^) nitrogen. Results suggested that the expression of *glnR* was activated in response to nitrogen starvation, and the *MsprpE* and four *Msacs* genes were further up-regulated at the transcriptional level (**Figure 1H** and **Supplementary Figure S1C**). Since propionyl-CoA and acetyl-CoA are both vital intermediates for SCFA assimilation, SCFA assimilation in *M. smegmatis* may be linked to nitrogen metabolism via the regulation by GlnR.

### Activity of MsPrpE and MsAcs Is Inhibited by MsPat-Mediated Acetylation/Propionylation

**FIGURE 2:**
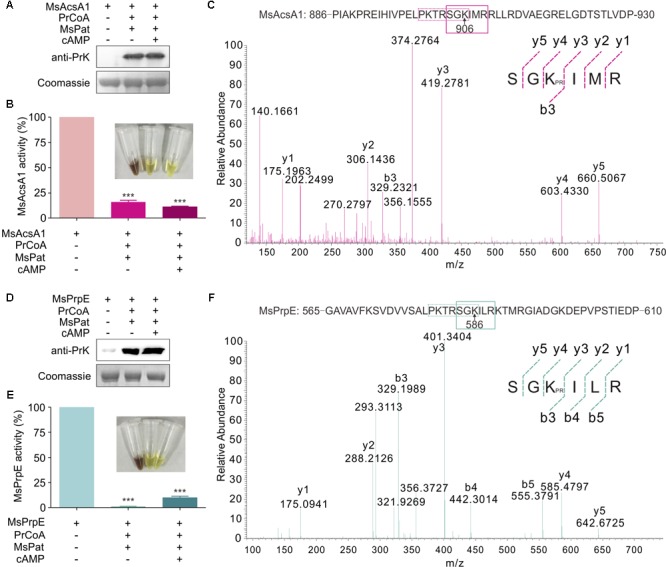
The activity of MsPrpE and MsAcsA1 are regulated by propionylation. **(A)** MsAcsA1 (2 μM) was incubated independently or with MsPat (2.7 μM), Propionyl-CoA (20 μM), and cAMP (1 mM) within a total volume of 100 μL at 37°C for 2 h, followed by SDS-PAGE analysis. The propionylation extent was verified using Western blot assay **(Upper)**. Meanwhile, another PAGE gel was stained with Coomassie brilliant blue as control **(Lower)**. **(B)** Enzymatic activity of MsAcsA1 using same condition with **(A)**. **(C)** LC-MS/MS spectrum of a tryptic peptide (m/z = 419.3) obtained from propionylated MsAcsA1. This spectrum matched the peptide sequence (boxed) in MsAcsA1, where a mass shift occurred between b3 and y4 ions consistent with propionylation at the lysine residue (amino acid position 906). **(D)** MsPrpE was incubated alone or in the presence of MsPat with the same condition of **(A)**, followed by SDS-PAGE analysis. The propionylation extent was verified by Western blot **(Upper)**. **(E)** Enzymatic activity of MsPrpE and MsAcsA1 using same condition with **(D)**. **(F)** LC-MS/MS spectrum of a tryptic peptide (m/z = 401.3) obtained from propionylated MsPrpE. This spectrum matched the peptide sequence (boxed) in MsPrpE, where a mass shift occurred between b3 and y4 ions consistent with propionylation at the lysine residue (amino acid position 586). Error bar represent the standard error, and all experiments replicate three times. ^∗∗∗^*P* < 0.001.

In a previous study, we found that Acs could be regulated by acetylation in *S. erythraea* (You et al., 2017). In addition, Acs in *Mtb* can also be regulated by reversible acetylation (Xu et al., 2011). We hypothesized that MsPrpE and MsAcs could be regulated by acylation (e.g., propionylation and acetylation) at the post-translational level. To directly validate this idea, we selected MsPrpE and MsAcsA1 for the propionylation and acetylation assays. Western blot analyses (**Figures 2A,D**) showed that MsAcsA1 and MsPrpE were propionylated by MsPat with propionyl-CoA (PrCoA) in the reaction system regardless of the presence of cAMP.

Many studies show PTM, such as propionylation and acetylation, play vital roles in the regulation of enzymatic activity (Xiong and Guan, 2012; Okanishi et al., 2014; Verdin and Ott, 2015). In this work, we found that the activity of both MsPrpE and MsAcsA1 were negatively modulated after propionylation, as MsPrpE was almost deactivated, whereas MsAcsA1 activity also decreased by more than 75%. Furthermore, the effect of propionylation on enzymatic activity was also evidenced by the change of color in (**Figures 2B,E**).

To identify the propionylation site(s) in MsAcs and MsPrpE, *in vitro* propionylated enzymes were separated by electrophoresis, and the bands corresponding to MsAcsA1 and MsPrpE were analyzed by LC-MS/MS. The results confirmed that a peptide with a sequence of SGKIMR (m/z = 419.3) contained a major propionylated lysine residue (Lys906) in MsAcsA1 (**Figure 2C**), and SGKILR (m/z = 401.3) with a propionylated lysine residue (Lys586) in MsPrpE (**Figure 2F**). Both lysine residues belong to the typical motif of PXXXXGK, which is recognized by bacterial acetyltransferases to acetylate the lysine residue in AMP-forming Acs (Starai et al., 2002). In the present study, we found that this motif could be recognized by acetyltransferases and subsequently acetylated and propionylated at its last lysine residue.

**FIGURE 3:**
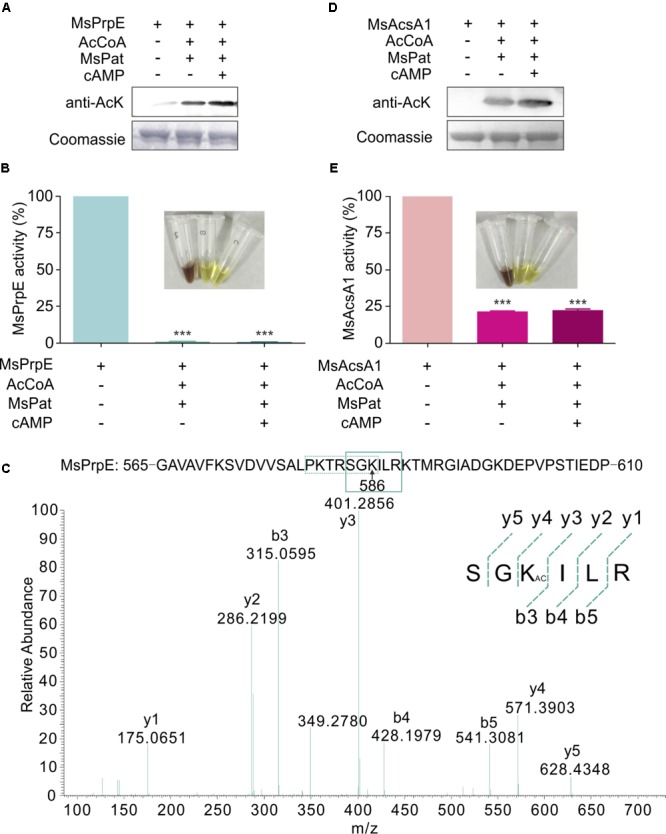
The activity of MsPrpE and MsAcsA1 are regulated by acetylation. **(A)** MsPrpE (2 μM) was incubated independently or with MsPat (2.7 μM), AcCoA (100 μM), and cAMP (1 mM) within a total volume of 100 μL at 37°C for 2 h, followed by SDS-PAGE analysis. The acetylation extent was verified using Western blot assay **(Upper)**. Meanwhile, another PAGE gel was stained with Coomassie brilliant blue as control **(Lower)**. **(B)** Enzymatic activity of MsPrpE using same condition with **(A)**. **(C)** LC-MS/MS spectrum of a tryptic peptide (m/z = 401.3) obtained from acetylated MsPrpE. This spectrum matched the peptide sequence (boxed) in MsPrpE, where a mass shift occurred between b3 and y4 ions consistent with acetylation at the lysine residue (amino acid position 586). **(D)** MsAcsA1 was incubated alone or in the presence of MsPat with the same condition of **(A)**, followed by SDS-PAGE analysis. The acetylation extent was verified using Western blot **(Upper)**. **(E)** Enzymatic activity of MsAcsA1 using same condition with **(A)**. Error bar represent the standard error, and all experiments replicate three times. ^∗∗∗^*P* < 0.001.

Western Blot also confirmed that in the presence of acetyl-CoA, MsPrpE and MsAcsA1 could be acetylated (**Figures 3A,D**). After acetylation, enzymatic activity sharply decreased as evidenced by disappearing colors, similar to the phenomenon observed in the propionylation assay (**Figures 3B,E**). The acetylation site in MsAcsA1 had been previously identified as SGKIMR (m/z = 419.3) using mass spectrometry (Xu et al., 2011), which was confirmed to be the same site (Lys906) in our propionylation analysis. In addition, LC-MS analysis suggested that the acetylation site (Lys586, SGKILR, m/z = 401.3) of MsPrpE coincided with the propionylation site (**Figure 3C**). Therefore, propionylation and acetylation could compete for substrates, thus affecting the relative concentration between propionyl-CoA and acetyl-CoA.

### GlnR Controls MsPrpE and MsAcs Acylation by Regulating Mspat Transcription

**FIGURE 4:**
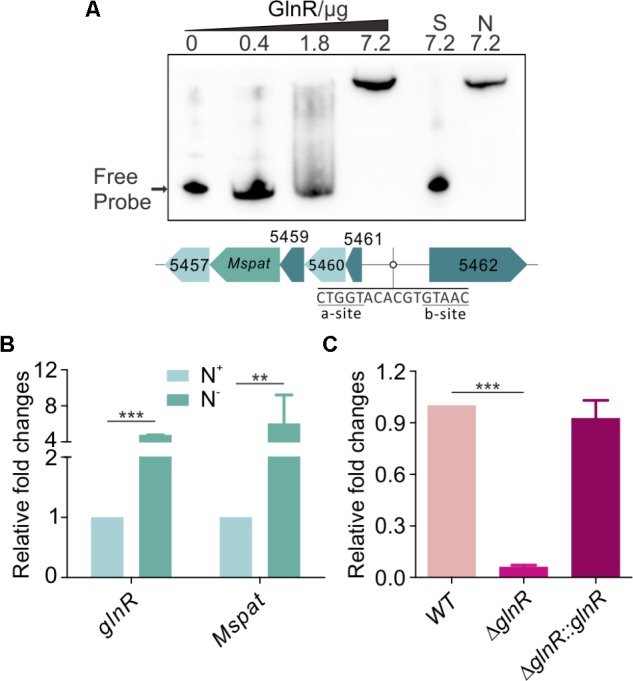
GlnR regulate the expression of *Mspat* in *M. smegmatis*. **(A)** EMSA identification of GlnR binding with the promoter region of *Mspat.* GlnR protein labeled with His tag with different gradient was incubated with probe (5 ng, Mspat). A 200-fold excess of unlabeled specific probe (S) or non-specific competitor DNA (sperm DNA) (N) were conducted as controls. **(B)** Comparative analysis of *Mspat* gene in the excess (N^+^) or limited (N^-^) nitrogen condition. **(C)** Differential expression analysis of glnR regulating *Mspat*. WT (wild type), *ΔglnR* (*glnR* knocked out mutant) and *ΔglnR::glnR* (*glnR* complementary) were cultured at 30°C to the middle exponential phase. Error bar represent the standard error, and all experiments replicate three times. ^∗∗^*P* < 0.01; ^∗∗∗^*P* < 0.001.

Our previous works have shown that GlnR regulates acetate metabolism and nitrogen metabolism in *S. erythraea* at transcriptional and post-translational levels (Liao et al., 2015; You et al., 2016, 2017). In this study, a classic motif (a-n6-b) was also identified upstream of *Mspat* promoter (**Figure 4A**). Therefore, *Mspat* could be mediated by GlnR in *M. smegmatis*. To verify this hypothesis, an EMSA assay was conducted with a gradient of GlnR concentrations. **Figure 4A** suggests that GlnR strongly and specifically binds to the regulation region of *Mspat*. As shown previously, the regulation of *MsprpE* or *Msacs* by GlnR is activated by nitrogen limitation. Here, we further demonstrate that the expression of *glnR*, which is closely associated with *Mspat* regulation, is also influenced by nitrogen availability.

**FIGURE 5:**
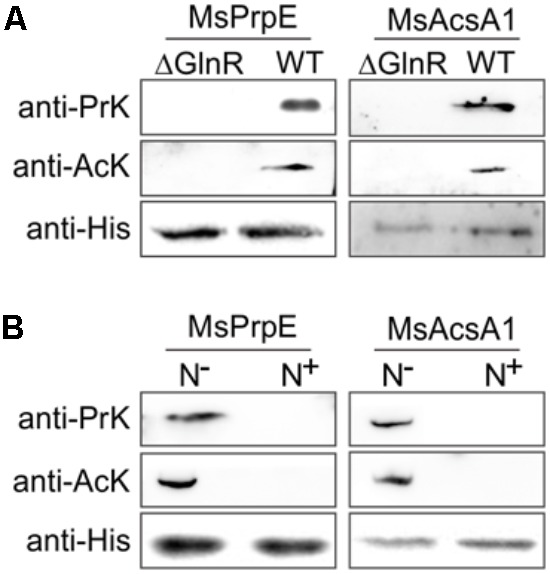
GlnR regulates acylation of the enzymes associated with short-chain fatty acid (SCFA) assimilation in *M. smegmatis*. **(A)** Equal His-PrpE or His-Acs protein (2 mg) purified from WT and ΔglnR strains of *M. smegmatis* cultured in LB medium were used to determine the propionylation level of MsPrpE **(Upper)** or acetylation level of MsAcs enzymes **(Middle)**. Equal non-acylation proteins were used as controls **(Lower)**. **(B)** Equal His-PrpE or His-Acs protein (1 mg) purified from *M. smegmatis* WT strain cultured in minimal Sauton medium with excess/limited nitrogen (N^+^/N^-^) were used to determine the Propionylation level of MsPrpE **(Upper)** or acetylation level of MsAcsA1 enzymes **(Middle)**. Equal non-acylation proteins were used as controls **(Lower)**.

As shown in **Figure 4B**, under nitrogen deprived conditions, expression of both *Mspat* and *glnR* was up-regulated, which indicates that *Mspat* could be modulated by GlnR under limited nitrogen conditions. To verify this hypothesis, we conducted a comparative analysis of *Mspat* in WT, *ΔglnR*, and *ΔglnR::glnR* strains (**Figure 4C**). Expression of *Mspat* decreased dramatically when *glnR* was knocked out, and was restored with *glnR* complementary. Therefore, the *Mspat* gene was regulated by GlnR in limited nitrogen environments. Considering that MsPat could propionate and acetylate MsPrpE and MsAcs (**Figures 2A–F, 3A–E**), we speculated that GlnR regulated the acylation of MsPrpE and MsAcs in response to nitrogen availability. The influence of GlnR on acylation level of MsPrpE and MsAcs was estimated by Western blotting using anti-propionated-lysine (anti-PrK) and anti-acetylated-lysine (anti-AcK) antibodies. As shown in **Figure 5A**, a sharper decrease in the propionylation and acetylation level of MsPrpE and MsAcsA1 was observed in *ΔglnR* compared to WT strains. The results demonstrated that the acylation of MsPrpE and MsAcsA1 were under the regulation of GlnR. As discussed, the expression of *glnR* is subjected to nitrogen availability. Western blot results in **Figure 5B** indicate that MsPrpE and MsAcsA1 could be acylated only under nitrogen deprived conditions, which suggest that the acylation of MsPrpE and MsAcs are under the control of GlnR in response to nitrogen signal in the environment.

### Effect of GlnR on the Growth and Viability of *M. smegmatis* in Macrophages

**FIGURE 6:**
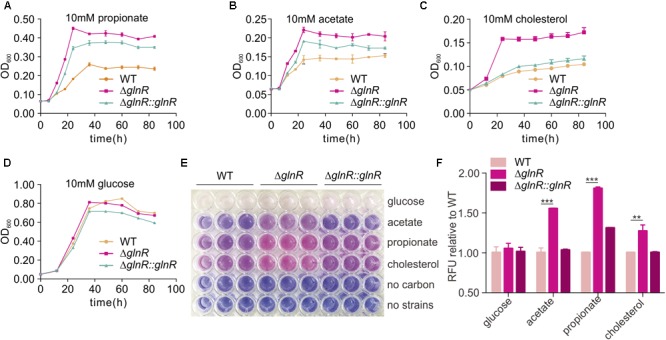
*glnR* gene mutation increase the growth and viability of *M. smegmatis*. **(A–D)** growth curves of the *M. smegmatis* WT, *ΔglnR* and *ΔglnR*::*glnR* strains growing in minimal medium with 10 mM propionate, acetate, cholesterol, or glucose, respectively. **(E)** the 96-well plate of 10 h incubation after resazurin addition. the metabolically active cells were indicated with pink color, whereas, the blue color represented resazurin reducing less and indicated low metabolic activity. **(F)** Quantification of resazurin fluorescence for cells growth. The fluorescence value at 10 h post-resazurin addition was normalized to the control of wild-type in different carbon condition. For **(A–D)** and **(F)**, error bars denote the SD of three replicates. ^∗∗^*P* < 0.01; ^∗∗∗^*P* < 0.001.

To investigate whether the regulation from GlnR on MsPrpE and MsAcs influenced nutrient utilization in *M. smegmatis, ΔglnR* and *ΔglnR*::*glnR* strains were further cultured on 10 mM propionate, acetate, cholesterol, and glucose, respectively. When *glnR* gene was deleted, growth on 10 mM propionate or acetate was higher compared to WT strains (**Figures 6A,B**), indicating an important role of GlnR in the assimilation of SCFAs in *M. smegmatis*. On the one hand, GlnR enhanced the transcription of *MsprpE* and *Msacs* (**Figures 1G,H** and **Supplementary Figure S1B**), as *glnR* deletion led to decreased enzyme activity of MsPrpE and MsAcs. Moreover GlnR up-regulated *Mspat*, resulting in increased acylation of MsPrpE and MsAcs. Acylation of these two enzymes decreased their activity, which further inhibited the utilization of nutrients including propionate, acetate in *M. smegmatis*. This regulatory network impacts the production of pigments (**Figures 2B,E, 3B,E**). Besides SCFAs, GlnR could also regulate the growth of *M. smegmatis* in cholesterol (**Figure 6C**), which is known as a vital carbon source for the survival of *M. smegmatis* in its host. Nevertheless, utilization of glucose in *M. smegmatis* was not affected by GlnR (**Figure 6D**).

Resazurin reduction assay was conducted to directly display the metabolic defects of *M. smegmatis* cultured with different SCFAs and cholesterol (Hayden et al., 2013). Resazurin as a blue compound which can be reduced by metabolically active cells. With resazurin reduced, it will be converted to a pink fluorescent substance which can be quantified to indicate the metabolic activity and cell number. For the resazurin test, target strains were cultured in a 96-well plate in propionate, acetate, cholesterol, or glucose-containing media. After 72 h of growth, resazurin was injected into each well and incubated for 10 h before visual examination of color. A significant decrease in the resazurin level was observed in WT, *ΔglnR* mutant, and complemented *ΔglnR*::*glnR* strains, as indicated by the pink color in **Figure 6E** and quantitated growth in **Figures 6A–D**. In propionate, acetate, and cholesterol media, the *ΔglnR* mutant strain reduced resazurin to greater extents compared to WT and *ΔglnR*::*glnR*. At the same time, the fluorescence of each strain was reported against that of WT in the same carbon source at 10 h after post-resazurin addition (**Figure 6F**). *M. smegmatis* metabolism was quantitated in different SCFAs and cholesterol as regulated by GlnR. In addition, in growth curve and resazurin analysis (**Figures 6A,C,F**), we found that the growth of *ΔglnR*::*glnR* strain is similar to WT in cholesterol as carbon, while it is different for *ΔglnR*::*glnR* strain growing in propionate. These results indicate that the amount of GlnR produced in complemented strain could be affected by different carbon. Phenotypic data demonstrate that GlnR responded to nitrogen level in the environment to regulate nutrient assimilation and utilization, which affected the viability and invasion of *M. smegmatis* in macrophage.

## Discussion

**FIGURE 7:**
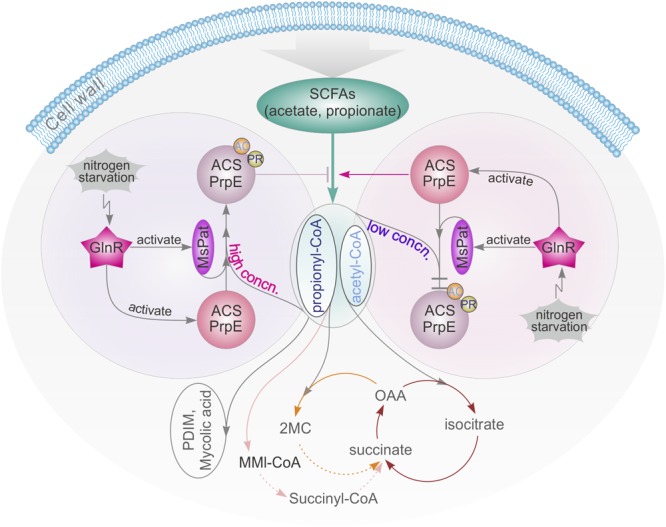
GlnR-mediated regulation of SCFAs assimilation revealed a tight connection of carbon and nitrogen metabolism in *M. smegmatis*. high concn., high concentration; low concn., low concentration; cycle with brown line: TCA cycle; cycle with yellow line: Methylcitrate cycle; cycle with pink line: Methylmalonyl CoA pathway; OAA, oxaloacetate; 2MC, 2 methylcitrate; MMI-CoA, Methylmalonyl-CoA; PDIM, phthiocerol dimycocerosate.

Carbon and nitrogen are crucial nutrients for *Mtb* survival. *Mtb* harnesses energy from the oxidation of various carbon sources (e.g., glucose, glycerol) to pyruvate and ultimately CO_2_ in central metabolic pathways. In macrophages, alternative carbon sources from the host, e.g., fatty acids and cholesterol, are utilized during *Mtb* infection (Schnappinger et al., 2003; Muñoz-Elías et al., 2005, 2006; Munoz-Elias and McKinney, 2005, 2006; Pandey and Sassetti, 2008). In this process, SCFAs such as propionate and acetate are critical intermediate metabolites (Starai et al., 2003). All catabolic pathways for propionate and acetate require activation of SCFAs into corresponding SCFAcyl-CoA forms before they can be converted and enter central metabolism to synthesize lipids that are essential for the survival and virulence of *Mycobacteria*, e.g., PDIM and mycolic acid (**Figure 7**). Nitrogen is another vital energy source, which exists as proteins, nucleic acids, and cell wall components. In bacteria, carbon and nitrogen are controlled by α-ketoglutarate (αKG), which represses enzyme I of the phosphotransferase system and in turn regulates the rate of glucose uptake in accordance with nitrogen level (Shimizu, 2013). In this study, it is found that the central nitrogen regulator, GlnR, can sense nitrogen shortages and up-regulate *MsprpE* and *Msacs* accordingly to increase the assimilation of SCFAs. GlnR can also up-regulate the expression of *Mspat* in nitrogen deprived environments and increase the acylation of MsPrpE and MsAcs to decrease the assimilation of SCFAs. On the one hand, under nitrogen limited conditions, GlnR activates MsAcs/MsPrpE to facilitate the assimilation of SCFAs to generate acetyl-CoA/propionyl-CoA and replenish the pool of acyl-CoA. The GlnR-mediated activation of acetyl-CoA synthesis under nitrogen-limited conditions leads to an increase of the intracellular concentration of nitrogen acceptor molecules (αKG and oxaloacetate), thus allowing for efficient assimilation of ammonium (You et al., 2017). Note this process is only in response to nitrogen starvation. On the other hand, the activation of MsPat leads to acylation of MsAcs/MsPrpE with acetyl-CoA/propionyl-CoA as a donor at the PTM level. This regulation is not only related to nitrogen signal, but also the level of intracellular acetyl-CoA/propionyl-CoA. If the concentration of intracellular acyl-CoA is high, PTM will be activated to inhibit the activity of MsAcs/MsPrpE and prevent further accumulation of acetyl-CoA/propionyl-CoA. In contrast, low concentration of acyl-CoA inhibits the acylation of MsAcs/MsPrpE, which remains active in SCFAs assimilation. Consequently, GlnR responds to nitrogen limitation to regulate SCFAs assimilation, whereas abundant nitrogen inactivates GlnR and inhibits SCFA uptake through TPM and reduced consumption of acyl-CoA through the acylation of MsAcs/MsPrpE (**Figure 7**).

Previous research has revealed that the metabolisms of carbon and nitrogen are interconnected (Fisher and Sonenshein, 1991; Wacker et al., 2003; Choi and Saier, 2005). The regulatory network of GlnR in different bacteria from the actinomycete group has also been extensively studied (Burkovski, 2007; Tiffert et al., 2011; Liao et al., 2015; You et al., 2017). In these bacteria, GlnR is activated at the nitrogen starvation status to regulate its target genes. The same mechanism has also been found in *M. smegmatis*, in which GlnR up-regulated *amtB, amt1, glnA*, and nirBD in response to nitrogen limitation (Jenkins et al., 2013; Gouzy et al., 2014). In this study, we extended the regulatory network of GlnR to assimilation of SCFAs in *Mycobacteria*, and demonstrated that GlnR acts as a bridge in a complex cross-talk regulatory network to maintain the homeostasis of carbon and nitrogen metabolisms (**Figure 7**).

The inability of many bacteria, including *Mycobacteria*, to assimilate propionyl-CoA inhibits their growth even in the presence of other carbon sources. This could explain why accumulation of intermediates often blocks other key pathways in these bacteria (Griffin et al., 2012): the accumulation of propionate or propionyl-CoA is toxic, thus needs to be controlled at a suitable level for detoxification. Previous work has shown that detoxification of propionyl-CoA relies on the activity of the methylcitrate cycle or the methylmalonyl pathway, or incorporation of propionyl-CoA into methyl-branched lipids in the cell wall (Lee et al., 2013). In addition, propionyl-CoA can also be used as a donor of propionyl groups for protein modification, which also provides an effective “sink” for excess propionyl-CoA (Singhal et al., 2015). In this study, we found that GlnR can further regulate *Mspat* to influence propionylation to control the amount of propionyl-CoA. In addition, MsAcsA1 and MsPrpE share the same acylation sites, which affects the balance between propionyl-CoA and acetyl-CoA, thus impacts propionyl-CoA removal. This result provides a new perspective in understanding the detoxification process in *Mtb*, which may benefit control of this pathogen in humans in the long run.

Besides SCFAs, *Mtb* mainly utilizes cholesterol as another carbon source from the host for survival during infection (Pandey and Sassetti, 2008). After internalizing into macrophages, *M. smegmatis* must sustain attacks from phagosomes (Anes et al., 2006), which makes assimilation and utilization of nutrients in macrophages vital for its survival. Here, it is found that *glnR* deletion (*ΔglnR*) can significantly increase the viability of *M. smegmatis* using cholesterol as the sole carbon source, though the roles of GlnR in cholesterol regulation remain unclear.

So far, the activation mechanism of GlnR remains unknown (Jenkins et al., 2013). Here, we found that there are different growth status for Δ*glnR*::*glnR* strain growing in cholesterol or propionate. These results indicate that the activation and production of GlnR in complemented strain may be impacted by different carbon due to *glnR* inserted in *M. smegmatis* genome as a locus other than it in WT.

As a model system of *Mycobacterium, M. smegmatis* shares many characteristics with its pathogenic relatives, including basic cellular processes and carbon metabolism features, which are complemented with additional pathways adapted to pathogenicity (Rastogi et al., 2001; Vissa et al., 2009; Chopra et al., 2014). We have found GlnR could directly bind with the regulatory region of *Mtbacs (Rv3667)* and *Mtbpat (Rv0998)* in *Mtb* (**Supplementary Figures S2A,B**). Therefore, GlnR could regulate *Mtbpat* and *Mtbacs* to influence the pathways of SCFAs assimilation in *Mtb*, in a similar mechanism to that found in *M. smegmatis*.

## Conclusion

We have established that the GlnR-mediated regulation of SCFA assimilation and cholesterol utilization in *Mycobacteria* is in response to nitrogen signal within the host. These findings could provide a deeper understanding of *Mtb* interaction with its host and its pathogenicity mechanisms for devising more effective control strategies of the pathogen.

## Author Contributions

X-XL, W-BL, and B-CY designed the research. X-XL and M-JS performed the experiments. All authors contributed to new reagents and analytic tools, analyzed data, and wrote the manuscript.

## Conflict of Interest Statement

The authors declare that the research was conducted in the absence of any commercial or financial relationships that could be construed as a potential conflict of interest.

## Supplementary Material

FIGURE S1 GlnR direct regulated *Msacs* genes. **(A)** EMSA identification of GlnR binding with the promoter region of *MsacsA2*, *MsacsA3*, and *MsacsA4*. **(B)** Differential expression analysis of glnR regulating *MsacsA2*, *MsacsA3*, and *MsacsA4*. **(C)** Comparative analysis of *MsacsA2*, *MsacsA3*, and *MsacsA4* gene in the excess (N^+^) or limited (N^-^) nitrogen condition.

FIGURE S2 GlnR (*Rv0818*) directly bind with the regulatory region of *Mtbacs* (*Rv3667*) **(A)** and *Mtbpat* (*Rv0998*) **(B)** in *Mtb*.
