# Supplementary figures and images for: GlnR-Mediated Regulation of Short-Chain Fatty Acid Assimilation in Mycobacterium smegmatis
Source: Front Microbiol. 2018 Jun 22;9:1311. doi: 10.3389/fmicb.2018.01311 (PMC6023979; doi:10.3389/fmicb.2018.01311)

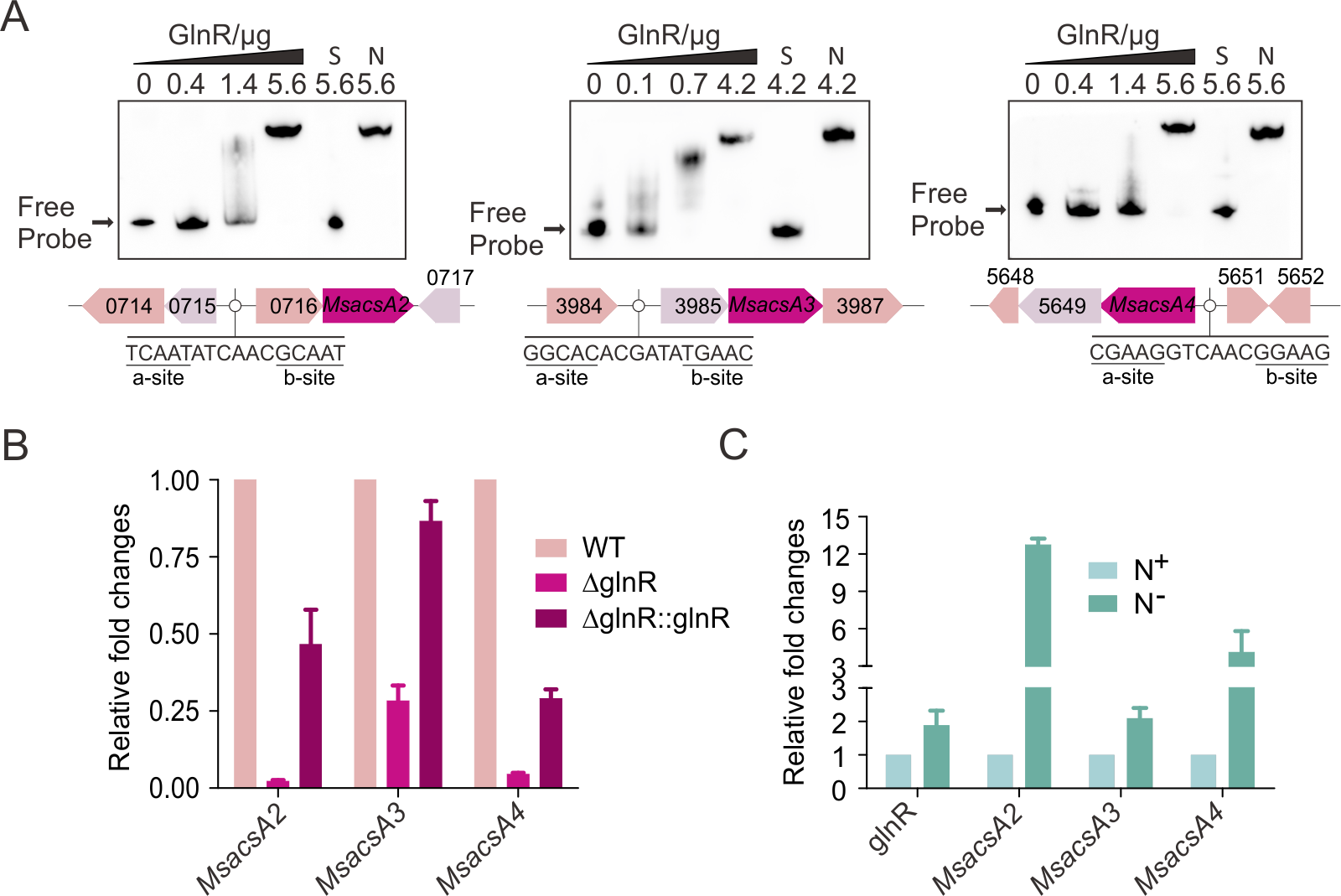

Supplement: FIGURE S1 — GlnR direct regulated Msacs genes. (A) EMSA identification of GlnR binding with the promoter region of MsacsA2, MsacsA3, and MsacsA4. (B) Differential expression analysis of glnR regulating MsacsA2, MsacsA3, and MsacsA4. (C) Comparative analysis of MsacsA2, MsacsA3, and MsacsA4 gene in the excess (N+) or limited (N-) nitrogen condition. [file Image_1.TIF]

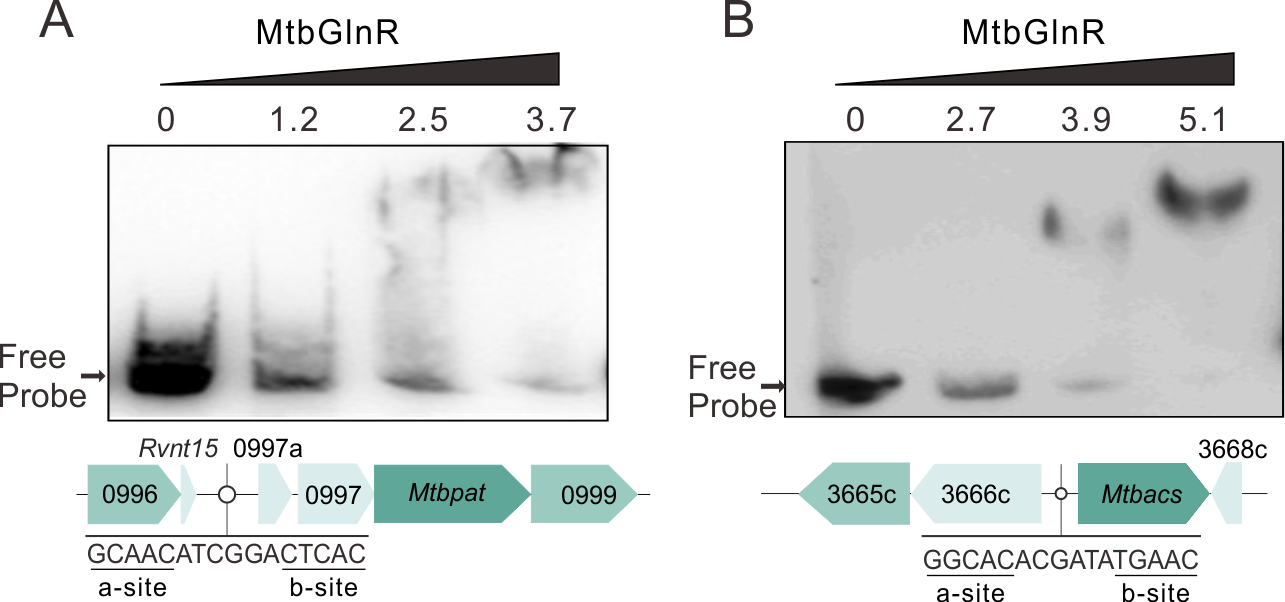

Supplement: FIGURE S2 — GlnR (Rv0818) directly bind with the regulatory region of Mtbacs (Rv3667) (A) and Mtbpat (Rv0998) (B) in Mtb. [file Image_2.TIF]
